# Fraternization

**Published:** 1859-02

**Authors:** 


### FRATERNIZATION.

Most strange affinities are taking place now-a-days, in the social, religious and political world, and not less in the world of literature. A missionary from the very far west writes, "I always read the Journal through, also Dr. Rice's 'Expositor' of Chicago, I cannot say as much of any other publication."

From the banks of the turbid Missouri, a lawyer of renown assures us, that he "expects" to take Hall's Journal of Health and the New York Observer as long as he lives. A note comes from one of the first divines in modern Athens, "whenever I receive the 'Journal' I read it through on the spot." A professional gentleman informs us, "There are two men's writings which I intend to have the very first moment of my ability, those of the Editor of the *Scalpel*, and Journal of Health." A Clergyman! writes us, "The Water Cure Journal, Life Illustrated, and Hall's Journal of Health ought to be in every family in the land." Another man thinks the *Independant* the best family paper extant, and his wife agrees with him! and further, that it and our Journal are indispensable to their comfort. Now if the JOURNAL pleases, and strikes the common sense of persons whose views so widely differ in the taking of other publications, the inference may be fairly drawn that it ought to have a circulation wider than either of them, and it would, if each of its friends would exhibit the same zeal in the promotion of what they feel to be useful and true, as the misguided advocates of error and false doctrine, show in their alacrity for the diffusion of the specious and the empty; but error is too often up and away by morning light, while laggard truth lies abed until breakfast. Gentle reader, resolve to break in upon this habit for one, by sending us the names of a dozen persons whom you love and esteem best, and thus serve truth and us too.

